# Neurotrophic Signaling Factors in Brain Ischemia/Reperfusion Rats: Differential Modulation Pattern between Single-Time and Multiple Electroacupuncture Stimulation

**DOI:** 10.1155/2014/625050

**Published:** 2014-07-14

**Authors:** Changpeng Wang, Fan Yang, Xiaoyan Liu, Ming Liu, Yun Zheng, Jingchun Guo

**Affiliations:** State Key Laboratory of Medical Neurobiology and Department of Neurobiology, School of Basic Medical Science, Fudan University, Shanghai 200032, China

## Abstract

Electroacupuncture (EA) treatment has been widely used for stroke-like disorders in traditional Chinese medicine. However, the underlying mechanisms remain unclear. Our previous studies showed that single-time EA stimulation at “Baihui” (GV 20) and “Shuigou” (GV 26) after the onset of ischemia can protect the brain against ischemic injury in rats with middle cerebral artery occlusion (MCAO). Here, we further investigated the differential effects between multiple EA and single-time EA stimulation on ischemic injury. In the present study, we found that both single-time EA and multiple EA stimulation significantly reduced MCAO-induced ischemic infarction, while only multiple EA attenuated sensorimotor dysfunctions. Also, with PCR array screening and ingenuity gene analysis, we revealed that multiple EA and single-time EA stimulation could differentially induce expression changes in neurotrophic signaling related genes. Meanwhile, with western blotting, we demonstrated that the level of glia maturation factor *β* (GMF*β*) increased in the early stage (day 1) of reperfusion, and this upregulation was suppressed only by single-time EA stimulation. These findings suggest that the short-term effect of single-time EA stimulation differs from the cumulative effect of multiple EA, which possibly depends on their differential modulation on neurotrophic signaling molecules expression.

## 1. Introduction

Stroke causes neurological injury and ranks the third mortality in the world. For several decades, interventions focusing on neuroprotection have been extensively explored in animal stroke models. However, safe and efficacious treatment strategies are still limited in clinic.

Acupuncture treatment is one of important therapeutic means in traditional Chinese medicine. It has been used to treat patients with stroke-like disorders for thousand years in China. In recent years, accumulating evidence from patients and experimental animals shows that acupuncture or electroacupuncture (EA) may initiate complex changes in neural-active genes and proteins in brain and exert protective effects against ischemic injury, as indicating a possible neural-related modulation effect of acupuncture in stroke treatment [1]. For instance, our previous work showed that single-time EA stimulation for 30 min at acupoints “Baihui” (GV 20) and “Shuigou” (GV 26) after the onset of ischemia could decrease extracellular glutamate level and increase extracellular taurine level after 60 min ischemia and the following 2 h reperfusion [2]. Such single-time EA application also induces brain protection [3–5] and expression changes of multiple factors [6–10] during acute ischemia/reperfusion. However, as a clinical therapeutic approach, application of multiple EA, rather than single-time EA, is mostly adopted to human subjects in a therapy session [11–13]. Unfortunately, although studies revealed that multiple EA improves neurological function in animal stroke models [14], there is less experimental evidence upon the underlying mechanisms of multiple EA treatment. Previous studies showed that multiple EA induces expression of Ang-1 and Ang-2 [15] and *δ*-opioid receptor [9] as well. However, whether multiple EA stimulation induces changes similar to those upon single-time EA stimulation? Delving into these questions will provide new evidence and shed new light in the area of acupuncture research. In this study, in order to clarify the differential effects between multiple EA and single-time EA stimulation on ischemic injury, we screened series of neurotrophin and receptors gene expression pattern in middle cerebral artery occlusion (MCAO) rat models and selected glia maturation factor *β* (GMF*β*) for further confirmation.

GMF*β* is a 17 KD peptide with 141 amino acids, first isolated and purified in 1989, and mainly expressed in the gliocyte and neuron [16, 17]. It is reported to induce the maturation of neurons and glias [18, 19]. Without the leading sequence, GMF*β* could not be secreted to the extracellular matrix [20]. Studies also demonstrated that GMF*β* can be phosphorylated by PKA, PKC, casein kinase II, and ribosomal S6 kinase (RSK) [21, 22] and inhibit the activity of Erk1/2 or enhance the activity of P38 [23]. Therefore, GMF*β* is considered to be involved in cellular signaling transduction pathways. In primary cultured astrocytes, knocking out GMF*β* leads to an enhancement in glutathione peroxidase and catalase activities, while overexpression of GMF*β* induces the expression of granulocyte-macrophage colony stimulating factor (GM-CSF) and cytokines production [16]. GMF*β* overexpression also aggravates the susceptibility of the renal proximal convoluted tubules toward the oxidative stress [24]. These studies indicated that GMF*β* might play important roles in the pathological processes of neurological diseases, such as stroke. Since there is no such research work of GMF*β* in stroke to date, in this study, we also investigated the expression change of GMF*β* protein both in ischemia and in the treatment of EA with single-time or multiple stimulations.

## 2. Materials and Methods

### 2.1. Animals and Experimental Group

Adult male Sprague-Dawley rats weighing 245 g–260 g were obtained from Shanghai Super—B&K laboratory animal Corp. Ltd. They were housed at 23 ± 1°C on 12 h/12 h light-dark cycle and permitted freely to food and water. After 3 days adaptation, they were randomly divided into sham-operation group, ischemia group, and ischemia plus EA group (IE Group). The total number of SD rats in this study was 186. All the experiments were approved by the Animals Care and Use Committees of Fudan University Shanghai Medical College, and all efforts were made to minimize the suffering of animals.

### 2.2. Induction of Transient Cerebral Ischemia

Transient middle cerebral artery occlusion model was performed according to the methods described by Longa et al. [25] with slight modifications. In brief, rats were anesthetized with 8% chloral hydrate (400 mg/kg, i.p.) and surgically exposed the left external carotid artery (ECA). After incision of ECA, a 4-0 monofilament nylon suture (0.26 mm diameter with a 0.34 mm diameter round silicone tip) was gently introduced from ECA into the left internal carotid artery up till the bifurcating origin of the left middle cerebral artery. After 60 min occlusion, reperfusion was accomplished by withdrawing the suture out of the ECA. For animals in the sham-operation group, the suture was advanced following similar procedures, except occlusion to the left middle cerebral artery.

In all the experiments, surgical duration was controlled within 15 min, and body temperature was maintained at 37 ± 0.5°C with a heating pad. We detected the blood pressure and blood gas before, during, and after the surgery to make sure that all the animals were in normal physiological conditions during experiments. In order to standardize the inclusive criteria, we monitored local cerebral blood flow at the surface of left parietal cortex (1.5 mm posterior to the bregma and 5 mm lateral to the sagittal suture) with a laser Doppler probe (Laser Doppler Flowmetry, Periflux System 5000, Perimed) [26]. MCAO animals that showed an immediate drop of ~80% in blood flow perfusion (of the preischemia baseline) were kept for further experiments and statistics.

### 2.3. Electroacupuncture Treatment

EA treatment was given 5 minutes after the ischemia onset. The acupoints are “Shuigou” (GV 26) and “Baihui” (GV 20). As described previously [26], these two acupoints are commonly used in clinical and experimental application and demonstrated to have benefits against brain ischemic injury. According to our previous study [4], in this work, EA was applied with stimulation parameters of 5/20 Hz at 1.0–1.2 mA by a therapeutic apparatus (G6805-II, Shanghai Medical Instruments High-Tech Co., China) and continued for 30 min. Single-time EA application was given only once after the ischemia onset, and the rats were sacrificed at day 1 after reperfusion, as reported by our previous studies [3, 4]. For the multiple EA application, EA was given once after the ischemia onset and additionally once a day for the next consecutive 6 days, and the rats were sacrificed at day 7 after reperfusion.

### 2.4. PCR Array Analysis

Three rats per group were used. Brain striatum was homogenized in TRIzol reagent (Life Technologies Corporation Invitrogen, USA) to extract the total RNA according to the manufacturer's instructions. RNA samples were treated with DNase I to remove the contaminating DNA and then purified with RNeasy MinElute purification kit (QIAGEN). cDNA was synthesized with SuperScript III Reverse Transcriptase (Life Technologies Corporation Invitrogen, USA). Gene assay was performed on the “GE array Q Series Rat Neurotrophin and Receptors Gene Array” (Super Array Bioscience Corporation), which includes 96 neurotrophic signaling genes involved in growth, differentiation, survival, and apoptosis. These genes are functionally grouped in 7 subsets: growth factors and receptors, apoptosis-related genes, chemokine receptors, cytokines and receptors, neuropeptides and receptors, neurotrophins and receptors, and signaling molecules. The measurement of each gene on the array was multiple for four times to get the mean datum. All signal intensities were normalized to the mean of housekeeping genes (*β*-actin, GAPDH, cyclophilin A, and ribosomal protein L13a). The effective threshold was set at 2.0 to identify genes of interest. Fold changes within 0.5–2.0 were considered that there is no change between the two groups.

### 2.5. Ingenuity Pathway Analysis

The ingenuity pathway analysis (IPA) application was used for further analyzing differentially expressed genes which were obtained from PCR array analysis. A detailed description is given in the online repository (http://www.ingenuity.com).

### 2.6. Determination of Infarct Volume

The rats were decapitated under anesthesia at determined time point after reperfusion, and rat brains were serially sectioned into 2 mm coronal slices. After being immersed in a solution of 2% triphenyltetrazolium chloride (TTC) for 5 min at 37°C, the slices were turned over and incubated for another 15 min and then transferred into 4% paraformaldehyde solution. The relative infarction ratio was calculated based on the following equation [10, 26, 27] to exclude the disturbance of ischemic-induced edema: (2 × nonischemic hemisphere's area − noninfarct area of whole brain slices)/(2 × nonischemic hemisphere's area) × 100%.

### 2.7. Western Blotting

The rats were decapitated under anesthesia at determined time point after reperfusion, and rat brains were quickly isolated and snap frozen. After being homogenized in a RIPA buffer containing protease inhibitor cocktail and centrifuged at 12,000 rpm for 20 min at 4°C, protein concentration was detected by Bradford reagent (Bio-Rad, Hercules, CA). The supernatants were then mixed with 2 × SDS-sample buffer, electrophoresed on SDS-PAGE gel, and then transferred onto polyvinyldifluoridine (PVDF) membrane. After being rinsed and blocked, the PVDF membrane was incubated with GMF*β* antibody (1 : 200, Santa Cruz, USA) at 4°C overnight and then HRP-labeled secondary antibody (1 : 4000, Chemicon, USA). The protein band was visualized with ECL Kit (Santa Cruz, USA). Protein levels were analyzed by integral optical density detection (Quantity One, Bio-Rad, Hercules, CA) and normalized to *β*-actin.

### 2.8. Neurobehavioral Test

The neurobehavioral evaluation was performed at days 1, 3, and 7 after reperfusion with corner turn test and foot-fault test described by previous studies [28, 29].

#### 2.8.1. Corner Turn Test

Rats were placed between two plastic boards (each with size of 40 × 60 × 1 cm^3^) which were adhered on the bottom board at the angle of 30° with a 5 mm width of opening at the joint to encourage the rats enter the corner. When entering the corner, the rats will turn back, and the turn was recorded as a left turn or a right turn. Each rat was recorded 10–12 times for each test before MCAO and during reperfusion [30]. The percentage of the number of times left turn was calculated. Turning movements without the vibrissae touching the side board were excluded.

#### 2.8.2. Foot-Fault Test

Rats were placed on an elevated horizontal ladder with 1 m in length, 10 cm in width, and 30 cm in height. The ladder rungs are 3 mm in diameter with a 2 cm gap between neighboring rungs. While moving along the ladder, rat right paw movement was recorded and scored according to the following criteria: Score 0: the right forepaw falls directly between the rungs, and the rat cannot keep forward; Score 1: the right forepaw slips between the rungs, and the rat cannot keep forward; Score 2: the right forepaw slips between the rungs, and the rat can keep forward; Score 3: the right forepaw slips from one rung to the adjacent rung to keep balance; Score 4: the right forepaw is placed not on the aiming rung but on an adjacent one; Score 5: the digits/toes or wrist/heel of the right forepaw is placed on the aiming rung; and Score 6: the midportion of the right forelimb is placed on the aiming rung. All the rats were given three-day training (three times per day) before the day of MCAO surgery and scored paw movement three trials for a test (the average value) before the onset of the surgery (baseline) and after reperfusion. The movement ratio was calculated as the total scores divided by the total step numbers. All ratios were then corrected by the ratio of baseline for further analysis.

### 2.9. Statistical Analysis

All data are presented as mean ± SD. The data of infarct volume were subjected to unpaired *t*-test. One-way analysis of variance (ANOVA) was used for neurobehavioral evaluation and western blotting. For all analyses, the changes were considered as statistically significant if the *P* value was less than 0.05.

## 3. Results

### 3.1. Reduction of the Ischemia-Induced Infarction by Single-Time or Multiple EA Stimulation

**Figure 1:**
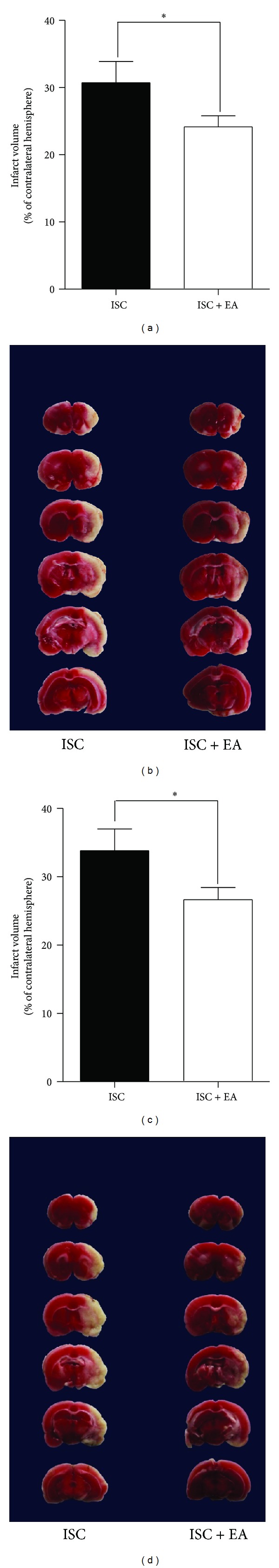
EA significantly reduced ischemia-induced cerebral infarction. TTC staining showed that after 60 min MCAO, the pale infarct is mainly located in the striatum and the frontoparietal cortex. When compared with ischemia group, both single-time EA (a, b) and multiple EA (c, d) decreased cerebral infarction. ISC: ischemia group; ISC + EA: ischemia plus EA group. *P < 0.05. *n* = 8 for each group.

In the ischemia group (*n* = 8), the infarct regions were mainly distributed in striatum and the frontoparietal cortex and enlarged from 30.7% ± 3.16% of the contralateral hemisphere at day 1 of reperfusion (Rep 1 d) to 33.8% ± 3.19% at day 7 of reperfusion (Rep 7 d, Figure 1). In the group of ischemia plus EA (*n* = 8), the infarction reduced to 24.2% ± 1.6% at Rep 1 d in single-time EA (*P* < 0.05, versus ischemia group) and 26.6% ± 1.79% at Rep 7 d in multiple EA (*P* < 0.05, versus ischemia group). In the sham operated group, no infarction was seen after reperfusion (data not shown). These results suggested that both single-time EA and multiple EA stimulation could attenuate ischemia-induced infarction.

### 3.2. Improvement in Neurobehavioral Test after Ischemia/Reperfusion by Multiple EA Stimulation

#### 3.2.1. Corner Turn Test

**Figure 2:**
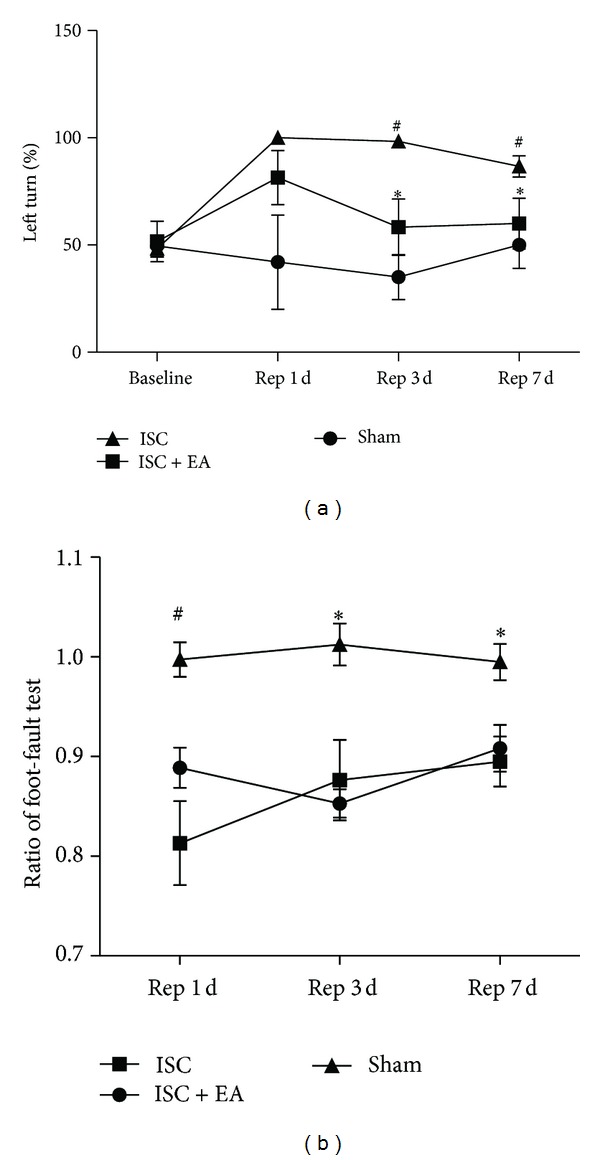
Multiple EA stimulation improved neurofunctional behaviors after ischemia/reperfusion. (a) Corner turn test performance. (b) Foot-fault test performance. Note that multiple EA treatment significantly reduced the left turn percentage in corner turn test at days 3 and 7 of reperfusion, whereas single-time EA stimulation had the tendency but no obvious effect in decreasing the left turn percentage (at day 1 of reperfusion). ISC: ischemia group. ISC + EA: ischemia plus EA group; Rep 1 d: day 1 of reperfusion; Rep 3 d: day 3 of reperfusion; Rep 7 d: day 7 of reperfusion. (a) *P < 0.05, ISC versus ISC + EA; ^#^ *P* < 0.01, ISC versus sham. (b) *P < 0.05, ^#^ *P* < 0.01. ISC versus sham. *n* = 6 for sham group; *n* = 8 for ISC group and ISC + EA group, respectively.

Before MCAO surgery, rats showed behavioral symmetries in corner turn test; namely, the left turn ratio was approximately equal to the right turn ratio (left turn baseline: 49% ± 4.8% in the sham group; 48.3% ± 4% in the ischemia group; and 51.7% ± 9.5% in the ischemia plus EA group, Figure 2(a)). After ischemia/reperfusion, animals subjected to the left MCAO showed a significant increase in the left turn percentage (98.3% ± 1.7% versus 35% ± 10.41% of sham operation, *P* < 0.01) at Rep 3 d. After 7 days of reperfusion, although the left turn percentage decreased to 86.7% ± 4.9%, it was still higher than that in the sham group (50% ± 10.8%, *P* < 0.01). In the group of ischemia + EA, multiple EA significantly reduced the left turns to 58.3% ± 13.1% at Rep 3 d (*P* < 0.05, versus the ischemia group) and 60% ± 11.8% at Rep 7 d (*P* < 0.05, versus the ischemia group). However, single-time EA did not decrease the left turns at Rep 1 d. These results indicated that multiple EA, but not the single-time EA, ameliorates the sensorimotor function after ischemia/reperfusion injury.

#### 3.2.2. Foot-Fault Test

As shown in Figure 2(b), there was a significant increase in the right forelimb foot faults after ischemia/reperfusion, which was expressed as a reduction in the test score ratio. Along with the extending of reperfusion time, the ratio increased from 0.813 ± 0.042 at Rep 1 d (*P* < 0.01, versus 0.997 ± 0.017 of the sham group) to 0.876 ± 0.04 at Rep 3 d (*P* < 0.05, versus 1.012 ± 0.021 of the sham group) and 0.895 ± 0.025 at Rep 7 d (*P* < 0.05, versus 0.995 ± 0.018 of the sham group). When compared with the group of ischemia, EA induced a slight but no significant increase in the test score ratio at the above reperfusion time points. No significant changes were observed between single-time EA (at Rep 1 d) and multiple EA stimulation (at Rep 7 d).

### 3.3. Differentiation in Gene Expression after Single-Time EA Stimulation or Multiple EA Stimulation

**Figure 3:**
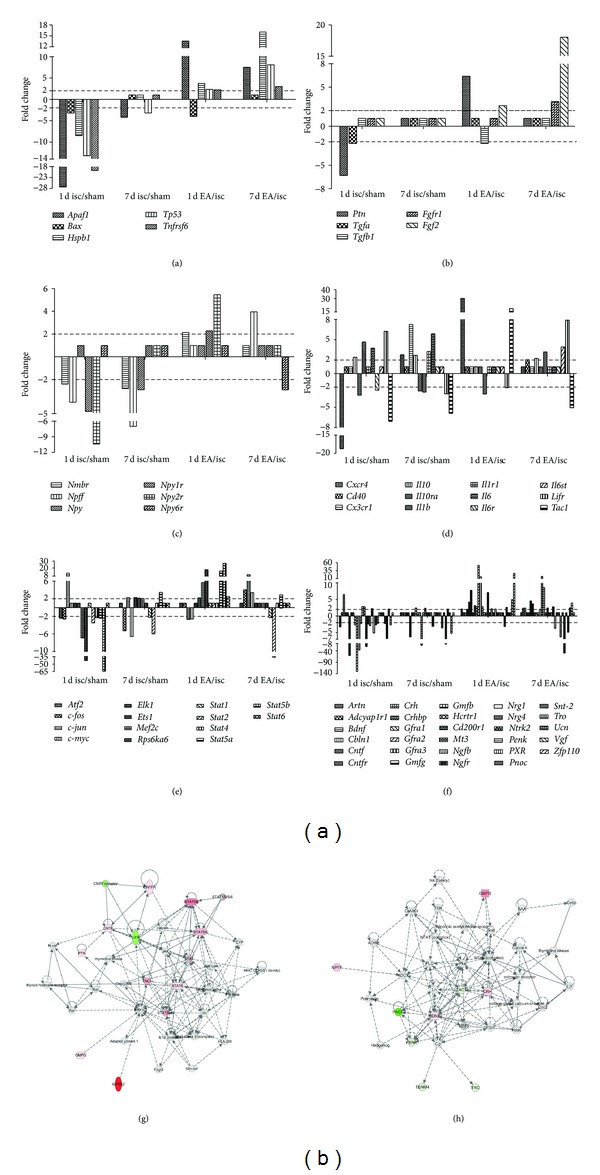
Gene expression change patterns in MCAO rats with or without EA stimulation at days 1 and 7 of reperfusion. The mRNA expression changes were determined by GE array Q series Rat Neurotrophin and Receptors Gene Array. Genes with remarkable expression change (greater than 2.0-fold) were grouped in apoptosis-related genes (a), growth factors and receptors (b), neuropeptides and receptors (c), inflammatory-related factors (d), signaling molecules (e), and neurotrophins and receptors (f). (g) (single-time EA stimulation) and (h) (multiple EA stimulation) show the further network analysis of differentially expressed genes by Ingenuity Pathway Analysis; red color means upregulated moleculars, green color means downregulated moleculars, and white color means the moleculars close functionally connected within the network predicted by IPA. isc, ischemia group; sham, sham-operation group; and EA, ischemia plus EA group. Rep 1 d, day 1 of reperfusion; Rep 7 d, day 7 of reperfusion.

Studies have found that striatal neurogenesis, accompanied with motor function improvement, is enhanced by exercise in MCAO rats [31], indicating gene expression alterations in ischemic striatum. Since we have found that multiple EA stimulation improves sensorimotor ability of ischemic rats, in this study, to further investigate gene expression changes in ischemic striatum under EA treatment, we detected the expression changes in mRNA level of a total 96 candidate genes (45 related to neurotrophins and their receptors, 10 related to neuropeptides and their receptors, 7 related to growth factors and their receptors, 10 related to cytokines and their receptors, 2 related to chemotactic factors and their receptors, 16 related to signal molecules, and 6 related to apoptosis-associated genes) at days 1 and 7 of ischemia/reperfusion with or without EA treatment. The results are shown in Figures 3(a)–3(f), Tables 1 and 2.

Our results showed that the expression levels of altogether 71 genes changed after ischemia/reperfusion with or without EA treatment. In the group of ischemia, when compared with the sham group, 10 genes were upregulated and 38 were downregulated at Rep 1 d (Figure 3, Table 1). When we took into account the functional grouping as described above, these upregulated genes belonged to chemokines/cytokines and receptors (IL10, IL1b, IL6, and LifR, Figure 3(d)), neurotrophins and receptors (Bdnf, Gmfb, Hcrtr1, Ucn, and Ngfr, Figure 3(f)), and signaling molecules (c-jun, Figure 3(e)). The downregulated genes belonged mainly to neurotrophins and receptors (Figure 3(f)).

At Rep 7 d, when compared with the sham group, 13 genes in the ischemia group were upregulated and 19 were downregulated (Figure 3, Table 1). Most of the upregulated genes belonged to chemokines/cytokines and receptors (Figure 3(d)) and signaling molecules (Figure 3(e)). As for the downregulated genes, there were 6 in neurotrophins and receptors (Figure 3(f)), 4 in chemokines/cytokines and receptors (Figure 3(d)), 4 in signaling molecules (Figure 3(e)), 3 in neuropeptides and receptors (Figure 3(c)), and 2 in apoptosis-related genes (Figure 3(a)).

Interestingly, when we compared these affected genes at Rep 7 d with those above (at Rep 1 d), 16 genes had similar expression change tendency (4 upregulation and 12 downregulation, Table 1(a)), whereas other 40 genes were in different expression change pattern (Table 1(b)). Among these 40 genes, 22 genes (10 belong to neurotrophins and receptors) showed decreased expression at Rep 1 d and no expression change at Rep 7 d. Five genes were downexpressed at 1 d but upexpressed at 7 d of reperfusion. Two genes expressions were upregulated at 1 d and back to the sham levels at 7 d of reperfusion. Three genes expressions were increased at 1 d but decreased at 7 d of reperfusion. Other eight genes showed no change at 1 d but notably change (4 decreased, 4 increased) at 7 d.

In the group of ischemia plus EA, when compared with the group of ischemia, the gene expression pattern changed differentially from that in the above groups (Figures 3(a)–3(f), Tables 2(a) and 2(b)), that is, 32 upregulated versus 7 downregulated at Rep 1 d (with single-time EA stimulation) and 27 genes upregulated versus 8 downregulated at Rep 7 d (with multiple EA stimulation). Among these EA-affected genes, 11 genes were upregulated at each of the reperfusion time points, while only one was downregulated at both of the two time points (Table 2(a)). The expression of other 44 genes changed in differential pattern (Table 2(b)): 17 genes showed no expression change at Rep 1 d but remarkable change (12 increased, 5 decreased) at Rep 7 d; 21 genes showed notably expression change (19 increased, 2 decreased) at Rep 1 d but no change at Rep 7 d; four were downexpressed at Rep 1 d and upexpressed at Rep 7 d; other two were upregulated at Rep 1 d and downregulated at Rep 7 d.

With IPA, we further organized the differentially expressed genes into networks (Figure 3(g), single-time EA stimulation; Figure 3(h), multiple EA stimulation). These results showed that there were differential expression change tendency and gene expression networks between single-time EA stimulation and multiple EA stimulation.

### 3.4. Single-Time EA Stimulation Reduced the Ischemic-Induced Increase in GMF*β* Expression in the Early Stage of Reperfusion

**Figure 4:**
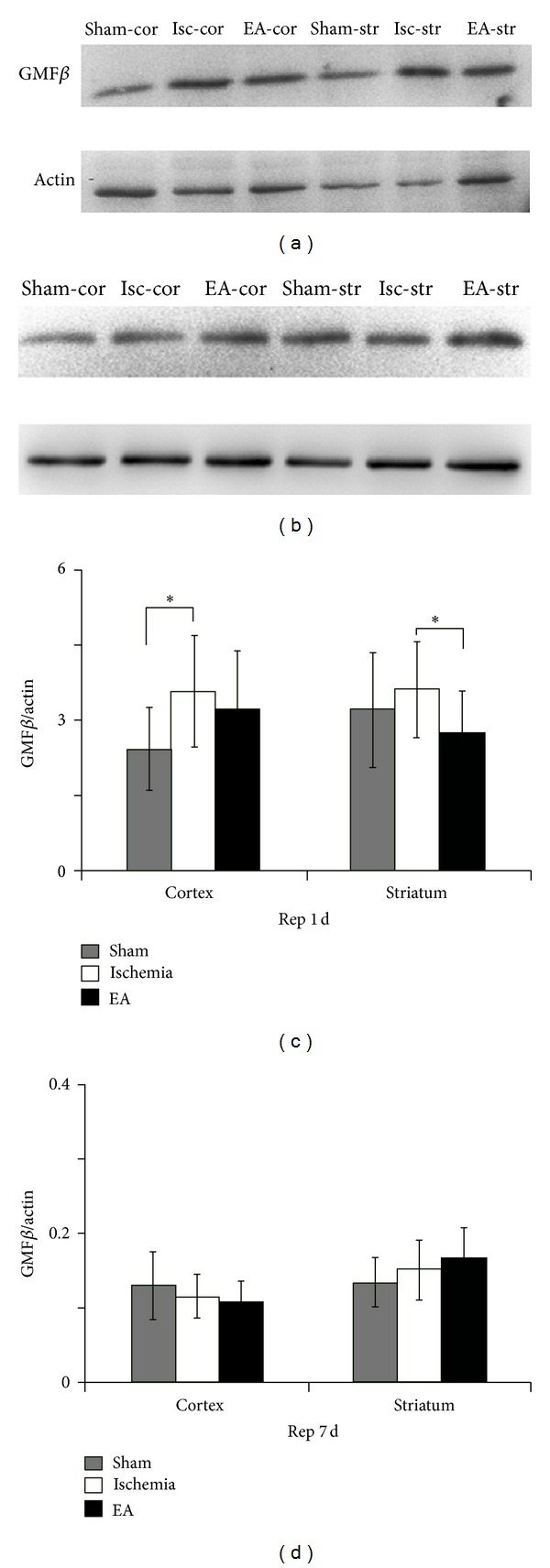
Western blot quantification of the expression of GMF*β* at days 1 and 7 of reperfusion. Representative images of western blot at day 1 (a) and day 7 (b) of reperfusion showed that GMF*β* was expressed in both striatum and cortex. The level of GMF*β* was notably increased in the ischemic cortex at the Rep 1 d (c). Note that single-time EA stimulation significantly reduced GMF*β* expression in the ischemic striatum (c), while multiple EA stimulation had no effect on GMF*β* expression at Rep 7 d (d). sham: sham-operation group; isc: ischemia group; EA: ischemia plus EA group; cor: cortex; str: striatum; Rep 1 d: day 1 of reperfusion; Rep 7 d: day 7 of reperfusion. *P < 0.05. *n* = 6 for each group.

According to the results of the Super Array screening, glia maturation factor *β* (gmf*β*) was one of the differentially expressed genes after EA stimulation (Figure 3(f), Tables 1(b) and 2(b)). Since up to now, there is no research report on GMF*β* function in ischemia; here, we further detected the protein levels of GMF*β* in ischemic striatum and ischemic cortex after ischemia/reperfusion with or without EA stimulation. Our results showed that, in the ischemia group, GMF*β* protein levels were significantly upregulated in the ischemic cortex (*P* < 0.05, versus sham group) and slightly increased in the ischemic striatum (*P* > 0.05, versus sham group) at Rep 1 d (Figures 4(a) and 4(c)), whereas they were almost kept unchanged at Rep 7 d (Figures 4(b) and 4(d)). EA stimulation made notably suppression of GMF*β* expression in the ischemic striatum (*P* < 0.05, versus ischemia group) at Rep 1 d with single-time EA (Figures 4(a) and 4(c)) but not at Rep 7 d with multiple EA (Figures 4(b) and 4(d)). These results suggested that single-time EA suppressed ischemia-induced GMF*β* levels at the early stage (1 d) of ischemia/reperfusion.

## 4. Discussion 

In the present work, based on the morphologic and neurobehavioral tests, we confirmed the protective effects of EA treatment against experimental stroke injury, which is consistent with our previous studies and others [1, 3–5]. Our results showed that multiple EA stimulation with specific parameters on GV 20 and GV 26 could significantly reduce the cerebral infarction and improve the sensorimotor ability of ischemic rats. Our findings also demonstrated for the first time that, during ischemia/reperfusion, multiple EA and single-time EA could differentially induce expression changes in genes of neurotrophins/receptors, growth factors/receptors, transcriptional factors, and inflammatory factors. Using Western blotting, we further measured the expression of GMF*β*, the protein encoded by one of the EA-affected genes gmf*β*, during ischemia/reperfusion with or without EA treatment.

In consistency with previous studies [3–5, 14, 32], our results confirmed that both single-time and multiple EA treatment protect MCAO rats against cerebral ischemic injury. Although the infarction volume was similar in both EA groups, our study provide the first evidence that multiple EA could induce better sensorimotor improvements than single-time EA. These results indicate that the short-term effect induced by single-time EA is not identical to the cumulative effect induced by multiple EA.

According to the gene screening analysis, our results suggest that the discrepancy between effects of multiple EA and single-time EA treatment is possibly due to their differential modulation on gene expression. As showed in Figures 3(b) and 3(f), the mRNA levels of growth factors/receptors and neurotrophins/receptors (such as Bdnf, Cntf, Fgf, Gdnf, Ngf, and their receptors) were mostly upregulated after one shot single-time EA and remained upregulated after multiple EA treatment; for example, Fgf2 and Fgfr1 mRNA expression remarkably rose after single-time EA and further increased after multiple EA (~18.1- and ~3.2-fold higher than those in ischemia group, resp.). For Bdnf, it was not changed in single-time EA but ~2.4-fold higher in multiple EA application than that in ischemia group. For Ngf and Ngfr, although Ngf levels only increased after one shot single-time EA, Ngfr was ~2.7-fold higher in multiple EA treatment. Even though GDNF receptor genes (Gfra1, Gfra2, and Gfra3) were mainly upregulated after single-time EA application, Gfra3 remained higher after multiple EA treatment. Therefore, although the infarction volume was similar in both EA groups, multiple EA application accumulates the enhanced effects on growth factors/receptors and neurotrophins/receptors expression and induces better sensorimotor improvements than single-time EA treatment. These results greatly replenish our previous knowledge [32–34] on the mechanisms underlying EA efficacy.

Furthermore, these results again provide strong evidence to the notion that the protective effect of EA against ischemic injury is through a multi-target-mediated network [1]. For instance, studies revealed that the family of IL-6 type cytokines includes IL-6, leukemia inhibitory factor (LIF), and CNTF. These neurotrophic cytokines share Il6st/gp130 as a common receptor subunit [35]. In our study, although Il6 and Il6r did not change after EA treatment, the mRNA levels of Lifr and Cntfr were notably increased after multiple EA treatment, as well as Il6st/Gp130 expression. These results suggest that the IL-6 type cytokines might play important roles in subacute or chronic phases of cerebral ischemia in multiple EA treatment.

Signal transducer and activator of transcription (STATs) are involved in several kinds of signaling pathway. Our results revealed that Stat5a, Stat5b, and Stat6 levels were remarkably increased after one shot single-time EA, whereas Stat1 and Stat2 were notably decreased after multiple EA treatment. Recent studies reported that STAT5 activation correlates with cardioprotection [36], which highly support our results of the Stat5a/5b upregulation after single-time EA. STAT1 and STAT2 are reported to be involved in signaling responses to interferon; their reduction in multiple EA treatment might contribute to anti-inflammation against the secondary injury after stroke. The differential expression pattern of Stat genes between single-time EA and multiple EA suggests that the downstream singling pathways in two types of EA application are not similar.

To confirm our results obtained from qPCR analysis, we further detected glia maturation factor beta (GMF*β*) protein expression. Studies reported that GMF*β* plays roles in oxidative-induced or inflammatory-induced cell death [16, 24, 37]. In our study, single-time EA reduced the expression of GMF*β* in striatum. Because GMF activation is found to be correlated with glial activation [38], EA application is also revealed to reduce ischemic-induced astrocyte activation [39]; therefore, single-time EA might suppress astroglial activation through downregulating glia maturation factor. We did not find significant differences among sham group, ischemia group, and EA group at day 7 after reperfusion. Since ischemia injury did not change GMF*β* levels in both cortex and striatum in chronic phase, it might be suggested that GMF is not closely related to inflammation in secondary injury after stroke.

In conclusion, the present study compared the effects of multiple EA and single-time EA stimulation on experimental ischemic stroke. Our results suggest that both single-time EA and multiple EA stimulation could modulate complicated gene/protein networks during ischemia/reperfusion and that the short-term effect of single-time EA stimulation differs from the cumulative effect of multiple EA.

## Figures and Tables

**Table 1a:** (a) List of genes which had similar expression change tendency at the two reperfusion time points

Functional groups	Gene	Rep 1 d (isc/sham)	Rep 7 d (isc/sham)
Apoptosis-related genes	Apaf1	↓↓	↓
	Tp53	↓↓	↓

Neuropeptides and receptors	Npff	↓	↓
	Nmbr	↓	↓

Inflammatory-related factors	Il10	↑	↑
	Il10ra	↓	↓
	Il6	↑	↑
	Tac1	↓	↓

Signaling molecules	Fos	↓	↓
	Jun	↑↑	↑
	Stat2	↓	↓

Neurotrophins and receptors	Cntfr	↓↓	↓
	Gmfg	↓	↓↓
	Hcrtr1	↑	↑
	Snt-2	↓	↓↓
	Vgf	↓	↓

**Table 1b:** (b) List of genes which had different expression change pattern at the two time points

Functional groups	Gene	Rep 1 d (isc/sham)	Rep 7 d (isc/sham)
Apoptosis-related genes	Bax	↓	—
	Tnfrsf6	↓↓	—
	Hspb1	↓	—

Growth factors and receptors	Ptn	↓	—
	Tgfa	↓	—

Neuropeptides and receptors	Npy	—	↓
	Npy1r	↓	—
	Npy2r	↓↓	—

Inflammatory-related factors	Cxcr4	↓↓	↑
	Cx3cr1	—	↑
	Il1b	↑	↓
	Il1r1	—	↑
	Il6r	↓	—
	Lifr	↑	↓

Signaling molecules	Atf2	↓	—
	Myc	—	↓
	Elk1	—	↑
	Ets1	—	↑
	Mef2c	↓	↑
	Rps6ka6	↓↓	—
	Stat1	—	↓
	Stat4	↓	—
	Stat5a	↓	↑
	Stat5b	↓↓	—

Neurotrophins and receptors	Artn	↓	—
	Bdnf	↑	—
	Gfra1	↓	↑
	Gfra2	↓↓↓	—
	Gfra3	↓↓	—
	Gmfb	↑	—
	Cd200r1	↓↓	—
	Mt3	↓	—
	Ngfb	↓	—
	Ngfr	↑	↓
	Nrg1	↓	—
	Nrg4	↓	—
	Ntrk2	↓	—
	Nr1i2	—	↓
	Tro	↓	↑
	Ucn	↑	—

**Table 2a:** (a) List of genes which had similar expression change tendency at day 1 and day 7 of reperfusion

Functional groups	Gene	Rep 1 d (EA/isc)	Rep 7 d (EA/isc)
Apoptosis-related genes	Apaf1	↑↑	↑
	Tnfrsf6	↑	↑
	Hspb1	↑	↑↑
	Tp53	↑	↑

Growth factors and receptors	Fgf2	↑	↑↑

Neurotrophins and receptors	Cntfr	↑	↑
	Gfra3	↑↑	↑
	Gmfg	↑	↑↑
	Cd200r1	↑	↑
	Pnoc	↓	↓↓
	Vgf	↑	↑
	Stat5a	↑↑	↑

**Table 2b:** (b) List of genes which had different expression change pattern at the two time points

Functional groups	Gene	Rep 1 d (EA/isc)	Rep 7 d (EA/isc)
Apoptosis-related genes	Bax	↓	—

Growth factors and receptors	Ptn	↑	—
	Tgfb1	↓	—
	Fgfr1	—	↑

Neuropeptides and receptors	Npff	—	↑
	Nmbr	↑	—
	Npy1r	↑	—
	Npy2r	↑	—
	Npy6r	—	↓

Inflammatory-related factors	Cd40	—	↑
	Cxcr4	↑↑	—
	Il10	—	↑
	Il1b	↓	↑
	Lifr	↓	↑
	Tac1	↑↑	↓
	Il6st	—	↑

Signaling molecules	Fos	—	↑
	Jun	↓	↑↑
	Myc	↓	↑
	Ets1	↑	—
	Mef2c	↑	—
	Rps6ka6	↑↑	—
	Stat1	—	↓
	Stat2	—	↓↓
	Stat5b	↑↑	—
	Stat6	↑	—

Neurotrophins and receptors	Artn	↑	—
	Adcyap1r1	—	↑
	Bdnf	—	↑
	Cbln1	↑	—
	Cntf	↑	—
	Crh	—	↑
	Crhbp	↑	—
	Gfra1	↑	—
	Gfra2	↑↑	—
	Gmfb	—	↑
	Ngfb	↑	—
	Ngfr	—	↑
	Nrg1	↑	↓
	Penk	—	↓
	Snt-2	↑	—
	Tro	—	↓
	Ucn	—	↑
	Zfp110	↑↑	—
